# Design of Experiments to Tailor the Potential of BSA-Coated Peptide Nanocomplexes for Temozolomide/p53 Gene Co-Delivery

**DOI:** 10.3390/pharmaceutics16111389

**Published:** 2024-10-29

**Authors:** Inês Afonso, Ana R. Neves, Dalinda Eusébio, Tânia Albuquerque, Eric Vivès, Prisca Boisguérin, Adriana O. Santos, Ângela Sousa, Diana Costa

**Affiliations:** 1 CICS-UBI—Health Sciences Research Centre, University of Beira Interior, 6200-506 Covilhã, Portugal; ines.sofia.afonso@ubi.pt (I.A.); a34643@ubi.pt (A.R.N.); dalinda.eusebio@ubi.pt (D.E.); tania.albuquerque@ubi.pt (T.A.); asantos@fcsaude.ubi.pt (A.O.S.); angela@fcsaude.ubi.pt (Â.S.); 2 PhyMedExp, Université de Montpellier, INSERM, CNRS, 34295 Montpellier, France; eric.vives@umontpellier.fr (E.V.); prisca.boisguerin@inserm.fr (P.B.)

**Keywords:** BSA-coated nanoparticles, WRAP-peptides, design of experiments, combined therapy, glioblastoma therapy

## Abstract

**Background:** Gene therapy can be viewed as a promising/valuable therapeutic approach directed to cancer treatment, including glioblastoma. Concretely, the combination of gene therapy with chemotherapy could increase its therapeutic index due to a synergistic effect. In this context, bovine serum albumin (BSA)-coated temozolomide (TMZ)-peptide (WRAP5)/p53 gene-based plasmid DNA complexes were developed to promote payload co-delivery. **Methods:** Design of experiments (DoE) was employed to unravel the BSA-coated TMZ-WRAP5/p53 nanocomplexes with the highest potential by considering the nitrogen to phosphate groups ratio (N/P), and the BSA concentration as inputs and the size, polydispersity index, surface charge and p53-based plasmid complexation capacity (CC) as DoE outputs. **Results:** The obtained quadratic models were statistically significant (*p*-value < 0.05) with an adequate coefficient of determination, and the correspondent optimal points were successfully validated. The optimal complex formulation had N/P of 1.03, a BSA concentration of 0.08%, a size of approximately 182 nm, a zeta potential of +9.8 mV, and a pDNA CC of 96.5%. The optimal nanocomplexes are approximately spherical. A cytotoxicity assay showed that these BSA-coated TMZ-WRAP5/p53 complexes did not elicit toxicity in normal brain cells, and a hemolysis study demonstrated the hemocompatibility of the complexes. The complexes were stable in cell culture medium and fetal bovine serum and assured pDNA protection and release. Moreover, the optimal BSA-coated complexes were able of gene transcription and promoted a significant inhibition of glioblastoma cell viability. **Conclusions:** The reported findings instigate the development of future research to evaluate their potential utility to TMZ/p53 co-delivery. The DoE tool proved to be a powerful approach to explore and tailor the composition of BSA-coated TMZ-WRAP5/p53 complexes, which are expected to contribute to the progress toward a more efficient therapy against cancer and, more specifically, against glioblastoma.

## 1. Introduction

Around ten million people die from cancer every year, making it the cause of around one in six deaths and one of the world’s biggest health problems. In this context, glioblastoma multiform (GBM) is the most lethal primary brain tumor, exhibiting a high rate of recurrence [1,2]. Although less prevalent when compared to other cancers, all GBM cases are classified as grade IV since there is still no truly effective treatment or cure. GBM is difficult to eradicate mainly due to its intrinsic localization and its aggressive profile. The first-line therapeutic approach includes surgery, followed by radiotherapy and/or chemotherapy, almost in all situations using temozolomide—a lipophilic alkylating drug [3,4]. Despite some increases in median survival, GBM remains one of the deadliest cancers worldwide [5]. This can drive from tumoral heterogeneity, the presence of glioma stem cells and treatment resistance, DNA damage repair mechanisms, and the blood–brain barrier—a biochemical and biophysical barrier that limits drug penetration and, thus, drug efficacy [6,7,8,9]. Altogether, these issues represent a difficulty for both researchers and clinicians, and the search for novel therapies is a priority. 

Gene therapy has had a great impact in the last decades and will continue to have a great impact in the research of innovative therapeutic modalities in various diseases, namely cancer [10,11,12]. Gene therapy, particularly focused on reestablishing p53 function, has been deeply investigated as a potential novel tool in cancer therapy [13,14,15,16]. p53 is a tumor suppressor protein involved in a multitude of roles (for instance, DNA repair, cell growth, cell metabolism, and immune response, to name a few) with a determinant role in keeping the genome integrity. This protein controls DNA repair by activating mechanisms such as apoptosis, cell cycle arrest, or senescence, depending on the level and type of DNA damage [17,18]. In this context, the functional activity of p53 is related to cell survival and cell cycle regulation. Being also present in the brain tissue, p53 interferes with several neural functions, and p53-associated signaling pathways are involved in many nerve injury processes [19,20]. Approximately half of all human cancers and 50% of GBMs bear mutations in the *TP*53 gene [21,22,23,24]. Mutant p53 loses tumor suppressor activity and displays an oncogenic structure. This oncogenicity inhibits apoptosis, leading to cancer cell survival and proliferation, contributing to tumor metastasis. In GBM, phenomena such as cell proliferation, invasion, or cell stemness have been linked to disrupted p53, and concretely, disruption of the p53-ARF-MDM2 pathway occurred to a great extent [25]. On the other hand, p53 exerts control over the expression of O-6-methylguanine-DNA methyltransferase (MGMT), a DNA repair enzyme responsible for the reversion of TMZ-induced DNA damage, tailoring glioma cells resistance to TMZ [25,26,27]. Moreover, p53 mediates the action of TMZ-independent apoptosis, promotes cell cycle G1 arrest, and controls the action of diverse transcriptional genes participating in DNA repair pathways [28,29]. In line with this, p53 supplementation in GBM tumors appears as an adequate strategy to induce efficient antiproliferation/death of cancer cells and to overcome TMZ resistance.

Nanotechnology has revolutionized a broad range of science fields, from chemistry to physics, biology, and so forth [30,31]. Gene therapy has strongly benefited from the exponential advances of nanotechnology. The design/conception of non-viral nanosystems suitable to load, protect, and release therapeutic payloads has promoted enhanced outcomes on drug/gene delivery mainly concerning targeting specificity, reduced cytotoxicity, release profile, and increased bioavailability, potentiating clinical translation [32,33,34].

The association of gene therapy with chemotherapy in pre-clinical models has brought benefits to cancer treatment due to synergistic effect [35,36,37]. The targeted and sustained/controlled co-delivery of anticancer drugs and the *TP*53 gene to cancer cells leads to the repression of their growth and expansion, circumventing secondary effects due to the application of lower drug doses, constituting a reliable approach to fight cancer. Cell-penetrating peptides (CPPs) are peptides containing no more than 30 amino acids and can load molecules of therapeutic interest [38,39]. Their facilitated translocation through the cell membrane, along with engineering tools to confer their targeting ability and reduced toxicity, make CPPs versatile and optimal candidates for integrated delivery nanocarriers for biomedical applications [40,41,42]. In the context of GBM therapy, CPP-based nanosystems showed to be promising as these carriers by deeply potentiating BBB penetration, tumor targeting, and TMZ effect [43,44]. Motivated by our previous research on the development of CPPs-based nanocomplexes to target and co-deliver payloads into glioma cells [45], in this work, we formulated BSA-coated peptide nanocomplexes for TMZ/p53 gene co-delivery. Albumin, an endogenous biomolecule, is non-toxic, biodegradable, and non-immunogenic. Moreover, albumin exhibits tumor-targeting capacity as it can bind to receptors overexpressed in tumor tissues, such as the cysteine-rich acidic secretory protein (SPARC) and gp60 [46,47]. Therefore, the incorporation of albumin in nanosystems is expected to enhance their delivery performance and therapeutic effect.

Design of experiments (DoE) has been recognized as a valuable statistical tool for both screening and optimization of formulations composition, and its utility in the development of “ideal” nano-drug delivery systems has been highlighted [48,49]. Following this, in the present report, DoE has been applied to reveal the optimal BSA-coated TMZ-WRAP5/p53 complexes for efficient co-delivery in glioblastoma therapy. Through the application of DoE, fundamental aspects of complex formulation have been addressed and unraveled, such as the ones regarding BSA coating and the parameters influencing complex properties. Thereafter, the optimal formulation achieved has been characterized concerning morphology, bio- and hemocompatibility profile, and stability in media. The optimal BSA-coated nanocomplexes displayed favorable properties for drug/gene co-delivery, encouraging in vitro studies of their application in the glioblastoma research context.

## 2. Materials and Methods

### 2.1. Materials

The pcDNA3-FLAG-p53 6.07 kbp plasmid was purchased from Addgene (Cambridge, MA, USA), amplified in a cell culture of E. coli DH5α, and purified using a methodology developed by our research group, as outlined in the literature [45]. WRAP5 was synthesized using a LibertyBlue^TM^; microwave peptide synthesizer (CEM Corporation, Matthews, NC, USA) with an additional Discover^TM^ module (CEM Corporation, Matthews, NC, USA) that combines microwave energy at 2450 MHz with the Fmoc/tert-butyl (tBu) approach. LC/MS (Waters, Saint-Quentin-en Yvelines, France) confirmed the peptide’s characteristics and purity, revealing a purity of 95% or superior. WRAP5 has an NH2-LLRLLRWWWRLLRLL-CONH2 sequence with 15 residues, an isotopic mass of 2104.34 g/mol, and 5 positive charges. TMZ was acquired from Frilabo (Lisbon, Portugal), BSA, sodium dodecyl sulfate (SDS), fetal bovine serum (FBS), and Triton X-100 were purchased from Sigma Aldrich Fine Chemicals Biosciences (St. Louis, MO, USA). Agarose and GreenSafe Premium were obtained from NZYTech (Lisbon, Portugal). Dulbecco’s Modified Eagle’s Medium (DMEM) high glucose with stable L-glutamine was obtained from Biowest (Nuaillé, France). A Milli-Q system from Millipore (Billerica, MA, USA) was used to purify water, and this ultrapure-grade water was used to prepare all the solutions.

The iQuant dsDNA HS Assay kit was purchased from ABP Biosciences (Rockville, MD, USA). The human normal astrocyte cell line, HA1800, isolated from a human brain (cerebral cortex) and cryopreserved, is from the American Type Culture Collection (ATCC, Manassas, VA, USA). U-87 human cells, a cell line isolated from malignant glioma from a male patient, likely with glioblastoma, were supplied by the European Collection of Authenticated Cell Cultures (ECACC, Salisbury, UK).

### 2.2. Methods

#### 2.2.1. Preparation of a Combined Aqueous Solution of TMZ and Peptide

The preparation of the TMZ-peptide mixture (TMZ-WRAP5) was carried out following a previously established protocol [45]. In summary, a solution of TMZ was prepared in ethanol at room temperature at a concentration of 0.5 mg/mL. Simultaneously, a 2 mg/mL solution of the WRAP5 peptide was prepared. Afterward, 150 μL of the peptide solution was mixed with 360 μL of the TMZ solution in 8 mL of water in a 15 mL falcon tube. An ultrasonic bath was used to disperse the solution for 90 s. The sample was frozen overnight at −80 °C and then lyophilized for 24 h. The sample was then dissolved in 200 μL of ultrapure water, and the peptide was quantified at 280 nm using a NanoPhotometer^TM^ (Implen, Inc., Westlake Village, CA, USA).

#### 2.2.2. Formation of TMZ-WRAP5/p53 Complexes

To form the TMZ-WRAP5/p53 complexes, 50 µL of TMZ-peptide solutions at the required concentration according to N/P ratio were added to 150 µL of pDNA solution (1 µg dissolved in Milli-Q water) drop by drop for 1 min under vortex agitation. The ratio N/P is defined as the molar ratio between the positively charged amine groups (N) of the peptide and the negatively charged phosphate groups (P) of the pDNA. After the formulation, the complexes were left to equilibrate at 25 °C for 25 min. The nanoparticles were then centrifuged for 20 min at 13,500× *g* at 4 °C. The obtained pellet was stored at 4 °C for up to two days before use.

#### 2.2.3. BSA Coating

A 20 mg/mL solution of BSA was prepared in ultrapure water and filtered to eliminate any potential impurities or large aggregates. The BSA concentration was determined by measuring the absorbance of the solution at 280 nm, considering the molar extinction coefficient of 43.824 M^−1^ cm^−1^. Using this BSA solution, diluted solutions were prepared ranging from 0.08 mg/mL to 0.28 mg/mL. BSA was incorporated into the complexes by two different procedures: (a) 37 μL of BSA solution was added before the complex formulation alongside the pDNA solution, here considered 0 min, and (b) 25 min after the complex was formulated (and before centrifugation).

#### 2.2.4. Agarose Gel Electrophoresis

The presence of non-encapsulated pDNA in the supernatant, after complexes centrifugation, was evaluated through electrophoresis for 40 min under 120 V in 1% (*w*/*v*) agarose gel, in 50 mL 1× TAE buffer (40 mM Tris base, 20 mM acetic acid, 1 mM EDTA at pH 8.0) and GreenSafe (0.6 µL) was used as a dye. For the visualization of the DNA bands on the gels, a Uvitec Fire-Reader system (Uvitec Limited, Cambridge, UK) was used.

#### 2.2.5. Design of Experiments (DoE)

DoE was used to optimize the formulation of BSA-coated TMZ-WRAP5/p53 complexes with the aim of minimizing the size of the complexes, maximizing the zeta potential, maximizing the pDNA CC, and maintaining an adequate PdI of less than 0.4 (outputs). Three factors were considered inputs: two numeric (namely the N/P ratio and the BSA concentration) and one categoric (BSA incorporation time). These inputs were studied at three levels (−1; 0; +1), and results from preliminary studies were considered to define the range. A central composite design (CCD) was applied, generating 22 experiments, considering 3 replicas of a central point. Statistical analysis was based on the use of Design-Expert version 11.

Equation (1) represents the equation of the generalized second-order polynomial model employed in the response surface analysis:

Y = β_0_ + β_1_X_1_ + β_2_X_2_ + β_3_X_3_ + β_12_ X_1_X_2_ + β_13_ X_1_X_3_ + β_23_ X_2_X_3_ + β_11_X_1_^2^ + β_22_X_2_^2^ (1)

After analyzing the suitability of the model applied to the DoE, the optimal point was determined and validated. In vitro assays were conducted using the conditions of the optimal point.

#### 2.2.6. Determination of Particle Size and Charge

Electrophoretic light scattering and dynamic light scattering at 25 °C were used to determine complexes’ zeta potential, average size, and PdI. The measurements were carried out using the Zetasizer Nano ZS device (Malvern Instruments, Malvern, UK). For this purpose, the pellet was resuspended in 5% glucose with 1 mM NaCl solution and placed in a disposable universal capillary cell. The size of the complexes was determined using a He-Ne laser at 633 nm with non-invasive backscattering (NIBS). The electrophoretic light scattering mode was employed to determine the zeta potential values using an M3-PALS (Phase Analysis Light Scattering) laser. The software Malvern Zetasizer v6.34 (Malvern Instruments, Malvern, UK) was used to obtain and process the data.

#### 2.2.7. pDNA Complexation Capacity

The pDNA CC of the nanocomplexes was evaluated using the iQuant^TM^ dsDNA HS Assay Kit. The iQuant^TM^ working solution was prepared by diluting the iQuant^TM^ dsDNA HS reagent in a ratio of 1:200 in 1× iQuant^TM^ dsDNA HS Buffer immediately before use. The prepared iQuant^TM^ working solution was then added (190 μL) to each well of a black 96-well clear bottom microplate (Greiner, Kremsmünster, Austria) to minimize fluorescence bleed-through. For the calibration curve, a series of standard dilutions of dsDNA were prepared. Subsequently, 10 μL of each dsDNA standard dilution and the supernatant of the nanoparticle samples in triplicate were added to the wells and mixed thoroughly by pipetting up and down. The microplate was then incubated for 2 min in the dark. Fluorescence was measured using the SpectraMAX Microplate Reader (Molecular Devices, San Jose, CA, USA), considering 485 nm as the excitation wavelength and 530 nm as the emission one.

#### 2.2.8. Scanning Electron Microscopy

The shape of the BSA-coated TMZ-WRAP5/p53 complexes was evaluated by Scanning Electron Microscopy (SEM). The complexes were formed and then centrifuged, and the resulting pellet was suspended in 40 μL of 2% tungsten (aqueous solution). This solution was then placed on a round coverslip and left to dry overnight at 25 °C. Subsequently, the samples were coated with gold using an Emitech K550 spray coater (London, UK). The complexes were then visualized using a Hitachi S-2700 scanning electron microscope (Tokyo, Japan), operating at an accelerating voltage of 20 kV at various magnifications.

#### 2.2.9. Cell Culture

HA1800 and U87 cells were cultured in 75 cm^2^ T-flasks with DMEM high glucose medium supplemented with stable L-glutamine (pH 7.45), 0.5 g/L sodium bicarbonate, 10% heat-inactivated fetal bovine serum (FBS), 1.10 g/L HEPES, and 0.1% (*v*/*v*) penicillin–streptomycin mixture (100 µg/mL each). HA1800 and U87 cells were maintained at 37 °C in a humidified atmosphere with 5% CO_2_ until reaching confluence.

#### 2.2.10. Evaluation of Cellular Viability

The effect on cellular viability displayed by the optimal BSA-coated TMZ-WRAP5/p53 complexes was monitored in HA1800 and U-87 cells using the colorimetric MTT (3-[4,5-dimethylthiazol-2-yl]-2,5-diphenyltetrazolium bromide) assay. First, 1 × 10^4^ cells per well were seeded in 96-well culture plates and maintained at 37° C and 5% CO_2_. BSA-coated TMZ-WRAP5/p53 complexes were added to serum-free DMEM medium (200 µL) and applied to the wells (0.1 µg of pDNA per well) for 4 h. After transfection, the culture medium was replaced with a serum-containing medium. After 24, 48, and 72 h of incubation with the complexes, cellular viability was assessed by the MTT reduction assay. For this, 20 μL of MTT solution with a concentration of 2 mg/mL was added to each well for 2 h. Thereafter, we removed the medium, and to each well was added 200 μL of DMSO for 30 min with shaking to enhance formazan crystals dissolution. The absorbance was measured using a Benchmark Microplate Reader (BioRad, Vienna, Austria) at 570 nm. The absorbance of the medium without cells was considered zero-absorbance. Non-transfected cells and cells treated with ethanol were considered positive and negative controls, respectively. In addition, cells transfected with TMZ and naked pDNA served as additional controls. The cellular viability was determined using Equation (2):

Cellular viability (%) = [A]test/[A]control × 100(2)

where [A]test is the absorbance of the test sample and [A]control is the absorbance of the positive control.

#### 2.2.11. Stability in Media

We studied the stability of optimal BSA-coated TMZ-WRAP5/p53 complexes after exposure to cell culture media with or without FBS supplementation. The aim was to replicate in vitro cell transfection conditions and simulate the extracellular environment of the human body. To conduct the test, nanocomplexes were centrifuged and suspended in 25 µL DMEM high glucose with stable glutamine, with and without supplementation with 10% FBS and streptomycin (1%)/penicillin (0.1%) antibiotics mixture solution, at 37 °C. BSA-coated TMZ-WRAP5/p53 complexes were then incubated at 37 °C for 4 h, and the release and degradation of pDNA were monitored using 1% agarose gel electrophoresis. A pDNA sample from stock solution (non-treated with serum or FBS) and naked pDNA (non-encapsulated) incubated with 25 µL DMEM high glucose with stable glutamine at 37 °C for 4 h were considered controls. In parallel samples, 1.5 µL of 10% SDS was added and incubated for 10 min to promote the decomplexation of pDNA from the nanocomplexes. Gel agarose electrophoresis (30 min under 120 V) was employed to analyze the samples.

#### 2.2.12. Hemolysis Test

To test the hemocompatibility of the optimal complexes, 2 mL of rat blood was collected in a heparinized tube supplemented with EDTA disodium salt. Subsequently, to separate the red blood cells (RBCs), the tube underwent centrifugation at 3000 rpm for 15 min at 4 °C. After centrifugation, the supernatant was discarded, and the RBCs were washed with a 0.85% *w*/*v* saline (NaCl) solution until clearness of the supernatant was achieved. Upon the washing process, a suspension of RBCs at a concentration of 3–5% was prepared in phosphate-buffered saline (PBS) with a pH of 7.4. A total of 900 µL of this RBC suspension was further incubated with 100 µL of the optimal BSA-coated TMZ-WRAP5/p53 complexes (1 µg of pDNA, N/P ratio of 1.03), each one resuspended in PBS pH of 7.4, for 1 h at 37 °C. For comparative analysis, PBS at pH 7.4 and Triton X-100 (1% solution) were employed as negative and positive controls, respectively. Upon conclusion of the incubation period, we made a centrifugation at 8000 rpm for 20 min. A UV–visible spectrophotometer (BioRad, Vienna, Austria) was employed to determine the absorbance at 576 nm, and the percentage of hemolysis was calculated considering the equation:

Hemolysis (%) = (Abs. Sample − Abs. Negative control)/(Abs. Positive control − Abs. Negative control) × 100(3)

#### 2.2.13. Gene Transcription in U-87 Cells

U-87 cells were seeded (1 × 10^5^ per well) in 12 well plates and cultured for 24 h in 1.5 mL of complete DMEM medium. After this period, the medium was replaced with an FBS and antibiotics medium free to optimize transfection. A total of 12 h later, complexes were added so that each well contained 1 µg of pDNA, and cells were left to incubate with complexes for 4 h. After 4 h incubation, the medium was replaced with complete DMEM medium again to stop the transfection process.

24 h after transfection, cells were collected for total RNA extraction. First, the culture medium was removed from wells, and cells were washed three times with PBS. Next, 300 μL of TripleXtractor (GRiSP, Porto, Portugal) was added and incubated at room temperature for 5 min. After incubation, the cell suspension is transferred to Eppendorf tubes, where 60 µL of chloroform is added. The mixture was vortexed for 15 s and incubated for *a* further 15 min at room temperature. The samples were then centrifuged at 12,000× *g* and 4 °C for 15 min. The upper phase, containing the RNA, was carefully transferred to new Eppendorf tubes, to which 150 μL of isopropanol was added. The samples were incubated and centrifuged again under the same conditions. The pellet formed was resuspended in 75% ethanol, followed by centrifugation for 5 min at 12,000× *g* and 4 °C. Finally, the RNA was resuspended in 20 μL of nuclease-free water and quantified using a NanoPhotometer^TM^. Additionally, the RNA was analyzed by 1% agarose gel electrophoresis. The cDNA was synthesized using the “Xpert cDNA Synthesis Kit” from GRiSP (GRiSP, Porto, Portugal), following the manufacturer’s protocol.

The p53 cDNA was amplified by conventional reverse transcription polymerase chain reaction (RT-PCR) method using the Speedy Supreme NZYTaq 2× Green Master Mix (NZYTech, Lisbon, Portugal), following the manufacturer’s instructions. The amplification reaction was prepared by mixing the necessary components in a 50 µL reaction volume. This included 25 μL of Speedy Supreme NZYTaq, 2× Green Master Mix, 1 μL of primer reverse (5′-CTG AGT CAG GCC CTT CTG TCTT-3′), and primer forward (5′−GAG CTG AAT GAG GCC TTG GA-3′) diluted in 1:20, 1 ng of cDNA and nuclease-free water to prefill the final volume. The PCR program was set up in a T100^TM^ Thermal Cycler (Bio-Rad Laboratories, Inc., Hercules, CA, USA), starting with an initial denaturation step at 95 °C for 5 min to ensure that DNA strands were fully denatured. This was followed by 30 cycles of amplification. Each cycle included denaturation at 94 °C for 2 s, annealing at 55 °C for 5 s, and extension at 72 °C for 5 s. A final extension at 72 °C for 2 min, after which the samples were held at 4 °C. PCR products were analyzed by electrophoresis and visualized in the Uvitec Fire-Reader system.

#### 2.2.14. Statistical Analysis

The statistical analysis involved a one-way analysis of variance (ANOVA) followed by the Bonferroni test. The obtained data are presented as the mean ± SEM based on at least three measurements. Data analysis was performed using GraphPad Prism V9.0.0 software. A *p*-value below 0.05 was considered statistically significant. Additionally, ** indicates *p* ≤ 0.01; *** indicates *p* ≤ 0.001; **** indicates *p* ≤ 0.0001.

## 3. Results and Discussion

### 3.1. Rationality of BSA-Coated TMZ-WRAP5/p53 Complexes

**Figure 1:**
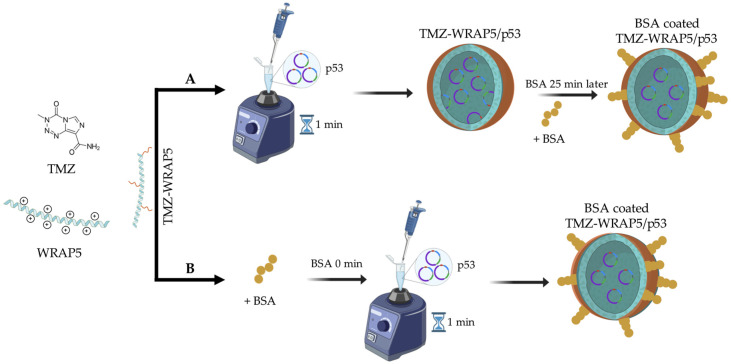
Illustrative scheme showing the formation of the complexes. (**A**) BSA coating 25 min after formulating the TMZ-WRAP5/p53 complexes; (**B**) BSA coating before formulating the TMZ-WRAP5/p53 complexes, considered 0 min.

The developed complexes are composed of a cell-penetrating peptide (WRAP5), TMZ, and plasmid DNA, leading to the formation of nanoparticles by co-precipitation induced by the electrostatic interactions between the positively charged amine groups of the peptide and the negative phosphate groups of pDNA. In the process, these complexes entrap and/or adsorb TMZ. The BSA was added to the TMZ-WRAP5/p53 complexes at two different times, 25 min post-formulation (Figure 1A) and before formulation—at 0 min (Figure 1B). These two different procedures were studied to unravel the influence of BSA into complexes formation and optimize the BSA coating process. BSA can bind to peptides at the complexes through various molecular interactions. The BSA hydrophobic regions facilitate the binding to the hydrophobic areas of the peptide. In addition, due to its negative charge, albumin can form ionic bonds with WRAP5 peptide, which has a positive charge of +5, or positively charged complexes [50].

Albumin-based complexes have been widely used due to their beneficial properties, such as biodegradability, lack of antigenicity, and surface modification to mitigate adverse drug toxicity [51]. In addition, albumin-based nanoparticles can break through the blood–brain barrier (BBB) and selectively target tumor cells through different mechanisms [52]. Tumor cells within glioma tissues exhibit heightened albumin uptake, relying on it as a vital amino acid and energy source [53]. To bolster albumin absorption within gliomas, there is an upregulation of secreted protein acidic and rich in cysteine, SPARC, an albumin-binding protein. Given the SPARC glioma cells overexpression, coating the complexes with albumin can be expected to improve tumor targeting and enhance specificity within this system [53,54]. This molecule has been extensively explored in the treatment of cancer with different types of nanoparticles, from iron oxide magnetic nanoparticles where the BSA-coated magnetic nanoparticles were formed as curcumin (CUR) carriers through desolvation and chemical co-precipitation for breast cancer treatment [55] or gold nanoparticles coated with BSA for cancer photothermal therapy [56].

TMZ-WRAP5/p53-based complexes have already been revealed to possess adequate characteristics that merit further study in the context of glioblastoma therapy [45]. In the present investigation, we explored the BSA coating of these TMZ-WRAP5/p53 nanocomplexes to enhance their non-immunogenic profile and lyoprotection, as the coating can function as stable shells. The optimization of the formulation process of BSA-coated TMZ-WRAP5/p53 complexes can, therefore, enhance their physico-chemical properties, which will contribute to their targeted and co-delivery performance toward glioblastoma.

### 3.2. DoE: Identification of Tuning Parameters

Lately, nanotechnology has emerged as an auspicious avenue for cancer therapy, mainly due to the ability of nanoparticles to serve as genetic and pharmacological vehicles [57,58]. It offers a range of benefits, such as enhancing existing therapies with superior targeting capabilities, boosting the effectiveness of localized drugs, and reducing systemic toxicity, among other assets [59]. Given the unique environment of tumors and their vascularization, nanosystems require specific characteristics. In general, ideal features of nanoparticles include a size ranging from 100 to 200 nm and a positive surface charge to promote cell internalization [54]. Furthermore, in the field of gene therapy, obtaining a high gene encapsulation rate is crucial for an effective therapeutic response. In this sense, it is clear that the rational design and optimization of the nanosystem properties are crucial steps to achieve high delivery and therapeutic performance.

DoE is a systematic method used in research and industrial processes to determine the relationship between factors affecting a process and the output of that process. DoE contributes to the planning, conduction, analysis, and interpretation of controlled tests to evaluate the factors that may influence a particular outcome or set of outcomes. The DoE application was considered to optimize the properties of BSA-coated TMZ-WRAP5/pDNA complexes for the simultaneous delivery of TMZ and TP53 genes for glioblastoma therapy. By exploring DoE tools, the main aim was to unravel how input/factors (alone and/or combined) affect the formulation properties (responses) [60]. Based on previous results conducted by our research group, which showed the ability of these complexes to form at low N/P ratio [45], here a range between N/P 0.5 and 1.5 was defined as input in the DoE

Concerning the BSA coating of the peptide complexes, initial analyses were carried out to establish a suitable range for the BSA. Complexes were prepared by adding BSA either before or 25 min after formulation in concentrations ranging from 0.05% to 0.5%. The supernatants were analyzed using agarose gel electrophoresis to evaluate the pDNA CC. The data available in the Supplementary Materials (SM), Figure S1, indicated that when BSA concentration exceeded 0.3%, the complexes were unable to fully incorporate the pDNA, resulting in concentrations ranging from 12.12 µM (0.08%) to 42.42 µM (0.28%), with the BSA coating before complexes formulation (at 0 min) or 25 min after formulation (Figure 1). Preliminary studies demonstrated that both the N/P ratio and BSA concentration, as well as the timing for the BSA coating process, are variables influencing the properties of TMZ-WRAP5/p53 nanocomplexes. Therefore, these parameters were taken as the inputs of DoE. The size, polydispersity index, surface charge, and pDNA CC were the selected properties as DoE outputs.

### 3.3. DoE Application: Finding the Optimal BSA-Coated TMZ-WRAP5/p53 Formulation

After establishing the inputs for the DoE, a CCD was implemented to optimize the formulation of TMZ-WRAP5/p53 complexes. This particular model is suitable for this work because it does not take into account points outside the range established for the inputs, which we already know in the preliminary studies that can lead to negative results due to the aggregation of nanoparticles [61]. Central composite models aim to estimate the non-linearity of response data and determine the curvature of continuous responses. They also make it easier to reduce the number of tests needed to estimate the squared terms in second-order models. In this way, central composite models have great value in experimental design and analysis [62,63].

Table 1 provides a comprehensive overview of all the runs suggested by the DoE, separated by BSA addition time—0 min or 25 min—(categoric input), with both N/P ratio and BSA concentration parameters listed in ascending order. To assess the reproducibility of the model, three central points (n = 3) were run under identical conditions, highlighted in gray in Table 1. The Design Expert software version 11 designed 22 experiments, and the respective outputs zeta potential, size, and PdI were revealed by electrophoretic and dynamic light scattering, and using the dsDNA HS Assay Kit determined the pDNA CC (%).

At BSA addition time zero, with the increase in BSA percentage in the formulation, we observed an increase in the size of the complexes but a decrease in complexes’ surface charge and, for most conditions, a decrease in pDNA CC. It was observed that increasing the concentration of BSA significantly increases the size of the complex. This fact can contribute to the higher size of the BSA molecule (7.1 nm and a molecular mass of 66.5 kDa) compared to the size exhibited by pDNA. This trend between the complex size and the BSA concentration has been previously documented [64]. Regarding the pDNA CC, the decrease in this parameter with BSA concentration may be due to the competition of electrostatic interactions with the peptide between BSA and pDNA [65,66]. Adding BSA at time 0 of the formulation can decrease the ability of the peptide to complex with the pDNA [67]. This phenomenon also leads to a more negative surface charge since BSA is negatively charged at physiological pH [68,69,70,71]. For instance, at an N/P ratio of 0.5, the nanoparticle size increases from 359.2 nm at 0.08% BSA to 431.84 nm at 0.28% BSA. Concurrently, the zeta potential becomes more negative, shifting from −17.3 mV to −31.6 mV. Similarly, at an N/P ratio of 1.0, higher BSA concentrations lead to an increase in nanoparticle size from 250.82 nm to 308.2 nm and a shift in zeta potential from a positive +3.8 mV to a negative −3.4 mV. The amount of BSA added to the complexes did not significantly alter the PdI, showing some uniformity among these conditions. These findings support the idea that BSA concentration can affect the characteristics displayed by complexes. The N/P ratio also seemed to influence the properties of the complexes, but in an opposite way when compared to the effect of BSA. Naturally, by increasing the N/P ratio, the charge of the complexes increases, becoming more positive, and the size decreases. At higher ratios, a greater amine content is present in the formulation, which strengthens the interaction between peptide and pDNA, leading to an increase in pDNA condensation. These conditions favor smaller complexes, more positive charges at the complex surface, and higher pDNA CCs [45]. We can see this behavior on the lowest N/P ratio (0.5) displaying sizes greater than 300 nm, specifically, 359.2 nm at 0.08% BSA and 431.84 nm at 0.28% BSA, compared to sizes below 300 nm for higher N/P ratios, such as 250.82 nm at an N/P ratio of 1.0 and 0.08% BSA. The PdI was also lower for high N/P ratios. The pDNA CC also reduces significantly when the ratio decreases, leading to significant differences within each addition time ranging from 35% (N/P 0.5) to 91% (N/P 1.5) at 0 min. This tendency of lower pDNA CCs at low N/P ratios was also found in a previous study by our research group, in which TMZ-WRAP5/pDNA complexes were formulated at different N/P ratios (0.1, 0.5, and 1) [45].

When BSA was added 25 min after formulation, the trend remained the same; increasing BSA concentration generally led to more negatively charged particles, larger sizes, and a less efficient pDNA CC. Higher N/P ratios yielded more positively charged particles with smaller size and higher pDNA CC values. As presented in Table 1, at an N/P ratio of 0.5, increasing the BSA concentration from 0.08% to 0.28% leads to a size increase from 311.25 nm to 350.13 nm, and the surface charge became more negative, changing from −12.4 mV to −25.7 mV. The pDNA CC also decreased significantly from 51.2% to 27%. At a BSA concentration of 0.08%, increasing the N/P ratio from 0.5 to 1.5 resulted in a significant decrease in particle size from 311.25 nm to 134.09 nm. The surface charge also shifted from −12.4 mV to 11.6 mV, and the pDNA CC improved dramatically from 51.2% to 95.3%. These phenomena are in line with the bibliographical information mentioned for time 0 [45].

Ultimately, the time of BSA addition proved to be a relevant input, generally altering the outputs. The complexes coated with BSA 25 min after formulation showed more favorable properties for payload intracellular delivery, such as more positive surface charges, decreased sizes, and lower PdI. At an N/P ratio of 1.0 and 0.18% BSA, the complexes size decreased from 299.5 nm at 0 min to 203.5 nm at 25 min, the zeta potential increased from +0.9 mV to +3.2 mV, and the pDNA CC improved from 78.3% at 0 min to 91.0% at 25 min. These complexes also generally displayed a lower PdI, such as decreasing from 0.344 at 0 min to 0.242 at 25 min, which reveals the uniformity in the particle size distribution. The main output indicating significant improvement with BSA coating at 25 min compared to the one at 0 min is the pDNA CC. This enhancement is corroborated by electrophoresis gel analysis of the supernatants of the BSA-coated TMZ-WRAP5/pDNA complexes (consult Figure S2, available in SM). The electrophoresis study demonstrates that by performing BSA addition at 0 min, the pDNA condensation is lower, as evidenced by the presence of pDNA in the supernatant. In contrast, by adding BSA 25 min after complexes formulation, pDNA is observed in the supernatant only for samples with lower N/P ratios, indicating improved pDNA CC.

Considering the goal of achieving BSA-coated TMZ-WRAP5/p53 complexes with higher surface charge, smaller size, a PdI between 0.2 and 0.3, and pDNA CC near 100%, it becomes notorious that complexes display favorable attributes when they are formulated at higher ratios (N/P 1 and N/P 1.5) and reduced quantities of BSA (0.08–0.18%) added 25 min after formulation.

Table 2 shows the multiple regression equations, the model applied to each output, and the statistical coefficients. The equations provided by the Design Expert analysis correlate the results with the different inputs, with an indication of positive or negative effect on the results by the sign underlying each factor [61]. With this approach, we achieved a deep understanding of the relationship between the independent variables and the respective results, highlighting their direction and effect. It is possible to analyze the influence of three key factors—N/P ratio (A), BSA concentration (B), and BSA coating time (C)—on four different responses: surface charge, size, PdI, and pDNA CC, as presented in Table 2. In the case of surface charge, significant positive effects of N/P ratio and BSA coating time were observed, along with a negative impact of BSA concentration. Increasing the N/P ratio results in a positive effect on the complex charge due to the greater amount of positively charged amino groups from the WRAP5 peptide [45]. On the other hand, increasing the amount of BSA increases the content of negative charges in the system, which has a negative effect on the overall charge of the BSA-coated TMZ-WRAP5/p53 complexes. These changes are evident in the variations in the size and charge of the nanoparticles outlined in the table. For example, at an N/P ratio of 0.5 and 0.28% BSA, the negative charge increases significantly to −31.6 mV while the particle size also grows to 431.8 nm. In contrast, at an N/P ratio of 1.0 and the same concentration of BSA, the charge is less negative (−3.4 mV), and the particle size is smaller (308.2 nm).

Similarly, size showed significant positive associations with both the N/P ratio and BSA coating time but a negative relationship with BSA concentration. The PdI was mainly influenced by the N/P ratio and BSA coating time, with negligible effects from BSA concentration. The pDNA CC showed positive relationships with the N/P ratio and BSA concentration, although with a lesser influence on coating time. However, it is evident that the influence of the input variables alters across the different outputs. Overall, the interaction of BSA coating time and N/P ratio always shows a positive effect on the four evaluated responses, while the interaction of the factors of N/P ratio and % BSA demonstrates a negative effect for all responses. These results of the factor interactions corroborate the influence of each independent factor and suggest that BSA coating time has a predominant positive influence.

The quadratic model was applied to all outputs. This model provides a robust framework for capturing nonlinear relationships between independent variables and the response, improving predictive capability across a broad range of input values. The quadratic model facilitates the identification of optimal experimental conditions, offering precision in achieving desired outcomes such as maximizing yield or minimizing defects [72]. The statistical coefficients obtained for each response are presented in Table 2 and were applied to unravel whether the statistical models generated from these experiments are valid and fit the data. The coefficient of determination (R^2^) provides insight into how well the regression model fits the data, and it should be close to 1 to evaluate if the model has high significance and the fitness of the output statistical model to the data [73]. The R^2^ values for the four outputs range from 0.8436 to 0.9841, suggesting the model fits the data. These values indicate that a proportion of the variability in the results can be attributed to the independent variables included in the model. Furthermore, the adjusted R^2^ values, which consider the number of predictors and penalize overfitting, remain high, ranging from 0.7474 to 0.9744. This suggests that the model explains the variability of the results, taking into account the complexity of the model [74]. The predicted R^2^ values reflect the predictive accuracy of the model, ranging from 0.7292 to 0.9371, indicating moderate to high levels of predictive ability of new data. The appropriate accuracy values represent the signal-to-noise ratio, suggesting variable levels of predictability between the results, with higher values suggesting the validity of the model in navigating the design space [74].

Table 3 shows the ANOVA analysis carried out to evaluate the significance of each factor and interaction term for the results of surface charge, size, PdI, and pDNA CC. This was achieved by comparing the variability of residuals of the current model with the variability between observations and replicate configurations of the factors. The results summarized in Table 3 demonstrated the statistical significance of the model for all outcomes, as indicated by p-values of less than 0.0001 for charge, size, and PdI and 0.0004 for pDNA CC. Factor A (N/P ratio) showed statistically significant effects on all results, with p-values of less than 0.0001. Factor B (BSA concentration) was significant only for charge and size outputs, and factor C (BSA coating time) was significant for all outputs except for pDNA CC. In the interaction terms AB, AC, and BC, only AB was significant for the charge output. The quadratic terms A^2^ and B^2^ exhibited mixed significance in the outputs. In addition, *p*-values of lack of fit were non-significant (>0.05) for all outputs. In conclusion, based on the results presented, the DoE model effectively fitted the data.

After understanding the impact of each input on the respective output, conducting statistical analysis on the coefficients, analyzing variance, and validating the statistical models of the DoE, the Design-Expert software predicted the optimum point for the BSA-coated TMZ-WRAP5/p53 nanocomplexes to maximize the pDNA CC and the positive surface charge, and at the same time to minimize the size and PdI. Table 4 presents the N/P ratio (A = 1.03), the amount of BSA (B = 0.08%), and the BSA coating addition time (C = 25 min) that is required to obtain the optimal formulation of the complexes, as well as the predicted outputs and a range of values for the validation of each output (95% of confidence interval). The predicted values for charge, size, PdI, and pDNA CC are +11.15 mV, 144.3 nm, 0.238, and 97.1%, respectively. Based on these values, the optimum point selected by DoE was tested in triplicate (n = 3), and the outputs were validated according to the data expected by Design-Expert software (Table 1). The optimal point revealed a zeta potential of +9.8 mV. This positive charge enhances cellular uptake, as the complexes can effectively interact with proteoglycan molecules (anionic) located on the cell surface [75]. The complexes presented a size of 182.3 nm, which is a beneficial size for cellular uptake. It is assumed that delivery systems displaying a size range of 100–200 nm are, by preference, internalized by cells and via endocytosis (clathrin- or caveolin-mediated processes) [76,77]. Optimal complexes have a PdI of 0.248 that shows a high degree of uniformity in the particle size distribution within a sample [78], and a pDNA CC of 96.5% ensures efficient pDNA loading/incorporation contributing to potential high gene expression and, therefore, enhanced therapeutic effect.

After the validation of the DoE, the properties of the same complexes without BSA, which served as a control for the following studies, were analyzed. The TMZ-WRAP5/pDNA complexes had a surface charge of +13.4 mV, a size of 140.7 nm, a PdI of 0.220, and a pDNA CC of 99.8%. The characteristics of the complexes without BSA coating allowed us to conclude that BSA was successfully inserted into the system. There was a slight decrease in the system’s charge due to the negative charge of the BSA and an increase in their size. DoE was revealed to be very useful in determining the role of BSA on the properties exhibited by the BSA-coated complexes and in determining the conditions at the formulation step that induces the conception of the “ideal” BSA-coated TMZ-WRAP5/pDNA complexes—the ones bearing the most favorable properties in terms of size, surface charge, PdI, and pDNA CC. In this sense, the DoE approach gave us control over the characteristics of the nanocomplexes formed, which may represent a great achievement in the design/development of co-delivery systems toward efficient glioblastoma therapy.

### 3.4. Study of Morphology

**Figure 2:**
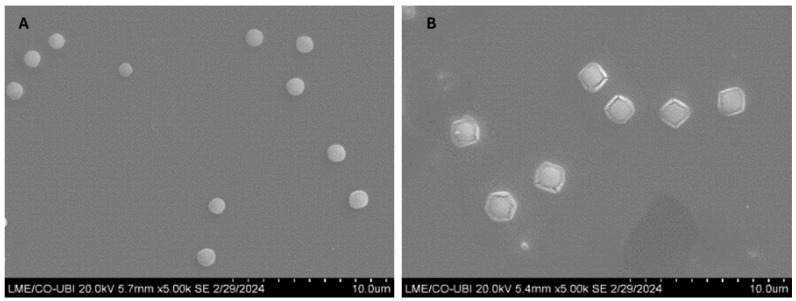
Scanning electron micrographs of BSA-free TMZ-WRAP5/pDNA complexes prepared at N/P ratio of 1.03 (**A**) and BSA-coated TMZ-WRAP5/pDNA complexes prepared at N/P ratio of 1.03 with 0.08% BSA (**B**). Scale bar = 5 µm.

SEM was considered to obtain knowledge into the morphology of the optimal point and the control, and the resulting images are shown in Figure 2. The BSA-free TMZ-WRAP5/pDNA complexes (A) had a spherical shape with a visibly smooth surface. On the other hand, the BSA-coated TMZ-WRAP5/pDNA complexes (B) exhibited a different morphology characterized by a more angular and textured appearance with a sectional view of a circle inside a squared or hexagonal-shaped coating layer. These results suggest that the BSA coating significantly influences the morphology of the TMZ-WRAP5/pDNA complexes and proves unequivocally that the coating was effective, with the BSA being probably located on the outside of the complexes.

We are aware that the sizes obtained from the SEM analysis are not in full agreement with the data from DLS. SEM allows for direct imaging of dry particles, and an alteration in the size of the complexes during the acquisition of micrographs is expected. DLS can be interpreted as an indirect assay for size determination as it is based on the intensity of molecular movement. It measures the hydrodynamic radii of the particles, which includes the particle itself but also the ionic and solvent layers associated with it in solution under the experimental defined conditions. DLS models the size from these data. Therefore, DLS data cannot be directly compared to dimensions provided by experiments carried out with other techniques. In this work, we considered DLS more reliable. However, at this stage, we must highlight that choosing a technique to characterize a nanosystem should take into consideration factors such as the expected size and the composition of the molecules, and various techniques, along with the accurate interpretation of data, must be advantageous.

### 3.5. The Optimal BSA-Coated Complexes Display a Non-Cytotoxic Profile

**Figure 3:**
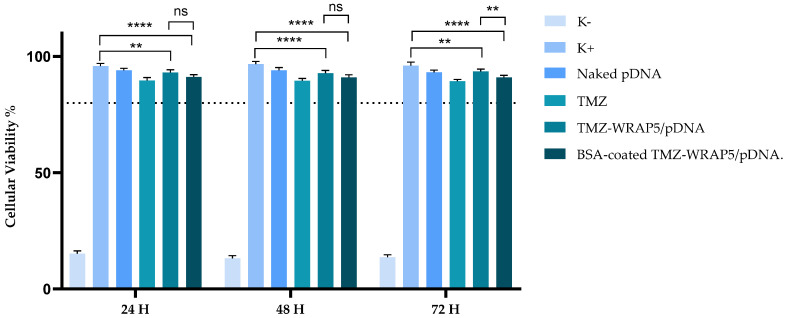
Cellular viability of HA1800 cells after 24 h, 48 h, and 72 h of incubation with the optimal BSA-free and BSA-coated TMZ-WRAP5/p53 complexes (0.1 µg of pDNA per well, N/P 1.03 and 0.08% BSA). Cells treated with ethanol were used as negative control (K−), and non-transfected cells were used as positive control (K+). Statistical analysis was completed using one-way ANOVA with data obtained from six independent measurements (mean ± SD, n = 6). (**** *p* ≤ 0.0001; ** *p* ≤ 0.01). ns—statistically non-significant.

Cytotoxicity analysis is an important step in the evaluation of the potential utility of delivery systems for biomedical uses. The toxicity displayed by nanosystems could severely limit their effectiveness in facilitating successful drug/gene therapy. To assess the biocompatibility profile of optimal BSA-coated TMZ-WRAP5/p53 complexes, an MTT assay was performed on human astrocytes at different incubation times (24, 48, and 72 h). The same experiment was carried out with the correspondent BSA-free complexes to reveal the impact of BSA coating on cellular viability. We used non-cancerous cells in this assay to demonstrate the biocompatibility of normal/healthy cells of the developed nanocomplexes before proceeding with assays to assess the therapeutic effect they might promote. Furthermore, the use of astrocyte cells provides insight into the specific interactions of the TMZ/p53 complexes with cells directly involved in glioblastoma disease. Also, by focusing on astrocytes, we were able to mimic the microenvironment of glioblastoma tumors. The results for the performed MTT assay are displayed in Figure 3. At 24 h, the pDNA-only complexes with an N/P ratio of 1.03 showed similar viability to the control cells, 94% and 93%, respectively, indicating their non-toxic profile. With the addition of 0.08% BSA, the viability of the cells was 91%, suggesting very low toxicity. In the 48 h and 72 h tests, the trend remained consistent. At 48 h, comparisons between the control cells and the complexes with an N/P ratio of 1.03 and with an N/P ratio of 1.03 and 0.08% BSA both show statistically significant differences (*p* < 0.0001) in cell viability, with viabilities of 93% for N/P 1.03, and 90% for N/P 1.03 with 0.08% BSA. The difference in viability between the N/P 1.03 complexes with and without 0.08% BSA was not significant (ns), indicating that the addition of BSA did not significantly alter the toxicity profile. At 72 h, the complexes with an N/P ratio of 1.03 exhibited a slight decrease in viability, from 95% to 93%, indicating a minor impact on cell viability (*p* = 0.056). The systems conceived considering an N/P ratio of 1.03 and 0.08% BSA led to a cellular viability of 91% (*p* < 0.0001 for the comparison with the control cells). The viability for the non-BSA-coated counterpart was 93%.

Although statistics indicated differences between the control cells and the TMZ-WRAP5/p53 complexes (for details, consult Figure 3), this study revealed that the optimal BSA-coated TMZ-WRAP5/p53 complexes do not induce any toxic effects on normal brain cells, as the obtained values for the cellular viability remained above 80%, what is in line with the non-toxic nature of the compounds based on ISO 10993-5 [79]. The preservation of astrocyte viability in the presence of these nanocomplexes suggests that they are non-toxic to normal brain cells, which is relevant to ensure the protection of healthy brain cells.

### 3.6. The Optimal BSA-Coated Complexes Are Stable and Protect pDNA

Evaluating the stability of BSA-coated TMZ-WRAP5/pDNA complexes in the extracellular environment and verifying the protection of the transgene is critical due to their significant impact on transfection success. To comprehensively investigate these aspects, we conducted protection and stability studies on optimal TMZ-WRAP5/pDNA complexes, both with and without BSA.

**Figure 4:**
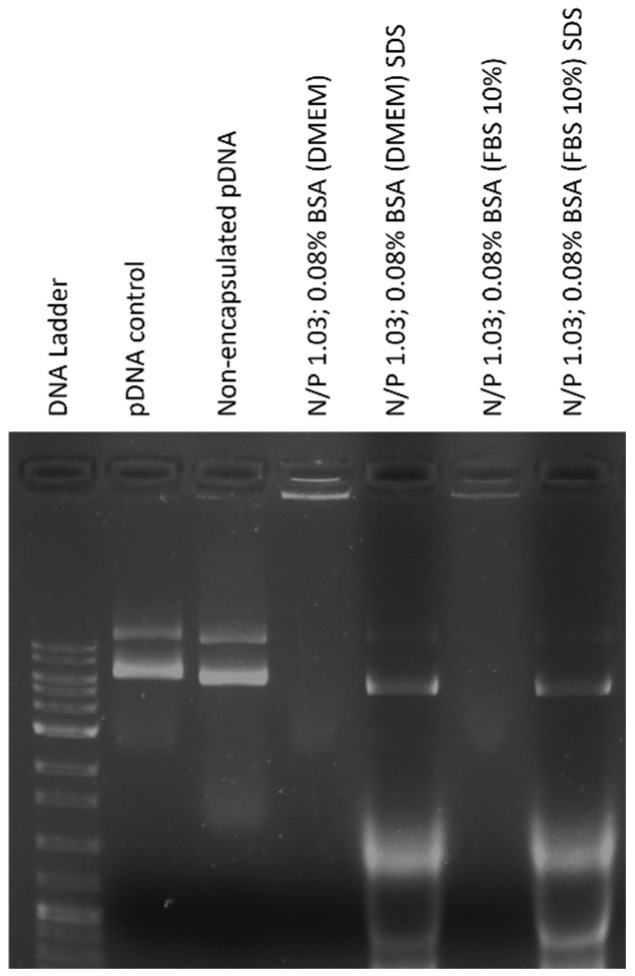
Electrophoretic analysis of the stability displayed by the optimal BSA-free and BSA-coated TMZ-WRAP5/p53 complexes after 4 h of incubation with DMEM high glucose with stable glutamine medium with 10% FBS or only 10% FBS. Lane 1: pDNA Molecular Weight Marker; Lane 2: pDNA sample from stock solution (non-treated with serum or FBS); and Lane 3: naked pDNA (non-encapsulated) incubated with 25 µL DMEM high glucose with stable glutamine at 37 °C for 4 h. The pDNA decomplexation was promoted by adding SDS and incubating for 10 min.

The results presented in Figure 4 demonstrated the stability and integrity of pDNA during a 4 h incubation period, as indicated by the absence of any pDNA bands on the agarose gel for both BSA-coated and non-coated TMZ-WRAP5/pDNA complexes. The absence of bands in the gel indicates that the pDNA remained intact throughout the incubation process, highlighting the protective capacity of the developed complexes. In contrast, non-encapsulated pDNA (Lane 3) was completely degraded after its incubation with DMEM high glucose with stable glutamine for 4 h. The results also demonstrated that through decomplexation by SDS, the pDNA was effectively released from the complexes without any signs of degradation, showing a predominant supercoiled isoform after being released (Figure 4). The supercoiled isoform of pDNA is critical for gene delivery as it is the most bioactive form and the isoform that promotes greater gene expression, such as for the p53 gene [80]. This further confirms the stability and protective nature of the optimal BSA-coated TMZ-WRAP5/pDNA complexes. The observed stability ensures that the pDNA remains protected from enzymatic degradation and other destabilizing factors present in the biological environment [81]. This protection is vital for maintaining the therapeutic effectiveness of the delivered genetic material.

### 3.7. The Optimal BSA-Coated Complexes Are Hemocompatible

**Figure 5:**
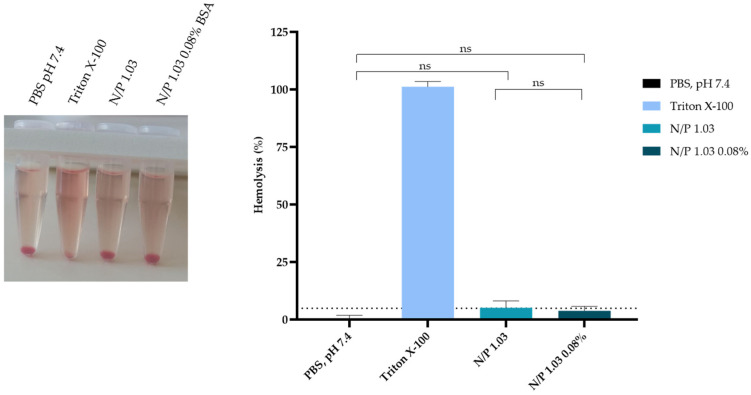
In vitro hemolysis assay was performed on rat red blood cells (RBCs), which were incubated with the optimal BSA-free and BSA-coated TMZ-WRAP5/p53 complexes (1 µg of pDNA, N/P ratio = 1.03). PBS pH 7.4 was considered the negative control, while in the positive control, RBCs were incubated with Triton X-100 (10%) to provoke hemolysis. ns—statistically non-significant.

The hemocompatibility of the optimal point, with and without BSA, was evaluated through a hemolysis test by incubation of TMZ-WRAP5/p53 complexes in rat blood cells. Figure 5 summarizes the obtained results. The nanocomplexes without BSA had a hemolysis rate of 5.9%, exceeding the threshold for non-hemolytic materials (5%). However, after adding BSA as a coating, the hemolysis rate decreased to 3.9%. BSA, being a stable protein compatible with the human body, minimizes the potential for triggering an adverse immune or inflammatory response that could contribute to hemolysis. None of the developed peptide nanocomplexes, however, exhibited statistically significant differences compared to the negative control, further reinforcing the absence of hemolysis.

These results demonstrated the non-hemocompatible behavior of the optimal BSA-coated TMZ-WRAP5/p53 nanocomplexes and, therefore, its biosafety for payload delivery applications.

### 3.8. The Optimal BSA-Coated Complexes Inhibit Glioblastoma Cell Viability

**Figure 6:**
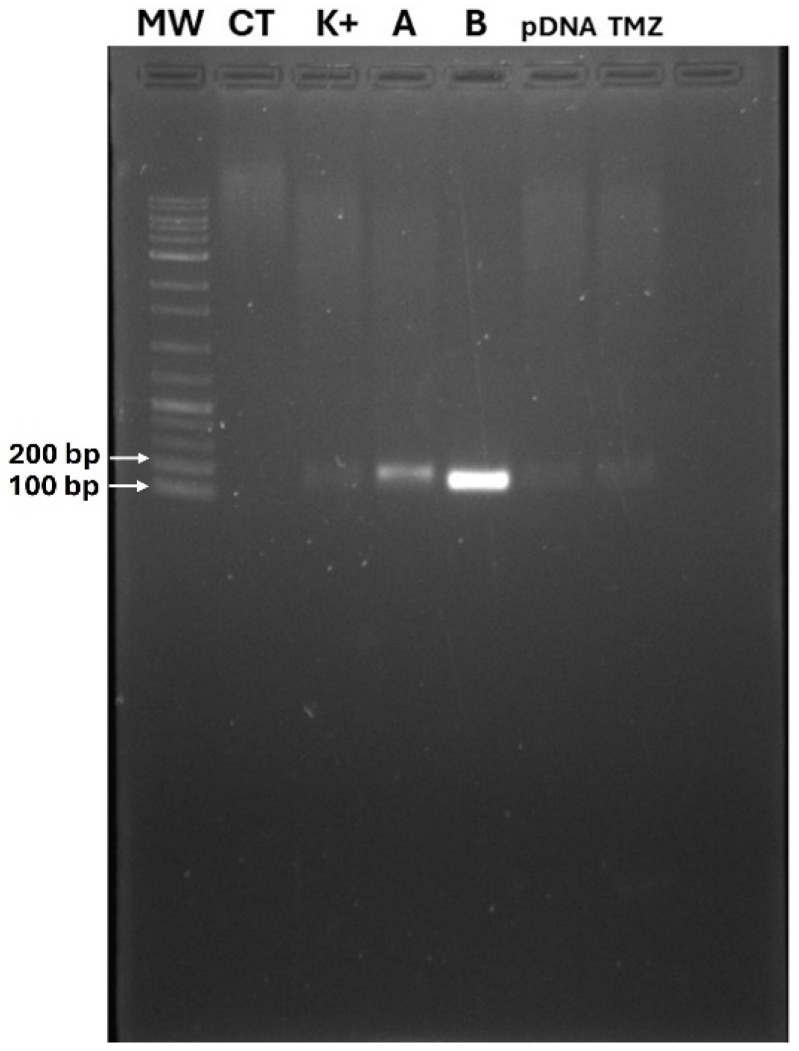
PCR analysis of p53 mRNA in U-87 cells after 24 h of transfection mediated by A—optimal BSA-free and B—BSA-coated TMZ-WRAP5/p53 complexes (1 µg of pDNA, N/P ratio = 1.03). MW—DNA ladder molecular weight marker; CT—control without cDNA sample; K+—untreated cells. Naked pDNA and TMZ alone were used as additional controls.

**Figure 7:**
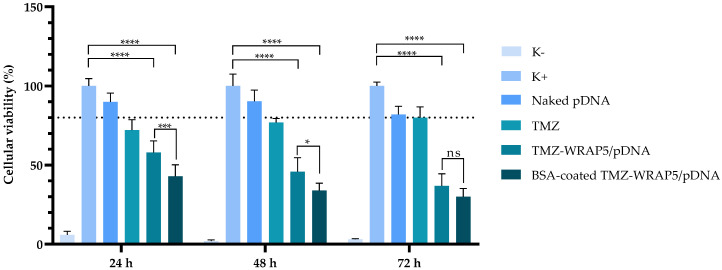
Cellular viability of U87 cells after 24 h, 48 h, and 72 h of incubation with the optimal BSA-free and BSA-coated TMZ-WRAP5/p53 complexes (0.1 µg of pDNA per well, N/P 1.03 and 0.08% BSA). Cells treated with ethanol were used as negative control (K−), and non-transfected cells were used as positive control (K+). Statistical analysis was completed using one-way ANOVA with data obtained from six independent measurements (mean ± SD, n = 6). (**** *p* ≤ 0.0001; *** *p* ≤ 0.001; * *p* ≤ 0.05). ns—statistically non-significant.

The p53 mRNA transcription in U-87 cells 24 h after transfection with the optimal BSA-coated TMZ-WRAP5/p53 complexes has been evaluated by RT-PCR assay with p53-specific primers. Non-transfected cells, cells transfected with naked pDNA, and cells transfected with TMZ alone were used as controls. The results are presented in Figure 6 and show significant levels of mRNA of p53 for both optimal BSA-free and BSA-coated TMZ-WRAP5/p53 complexes (band at 150 bp), demonstrating the efficiency of both complexes to induce gene transcription in glioblastoma cells. A more intense band from p53 transcripts can, however, be observed for the transfection carried out by the optimal BSA-coated complexes, suggesting higher levels of mRNA. After evidencing gene expression, the potential effect on the reduction in U-87 cell viability promoted by the BSA-coated TMZ-WRAP5/p53 complexes has been researched. The data from an MTT study performed at 24, 48, and 72 h post-transfection are summarized in Figure 7. Independently of the time considered, all samples exhibited statistically significant differences in comparison with the positive control, **** *p* ≤ 0.0001, demonstrating an induced effect on the viability of U-87 cells. The reduction in the cellular viability is particularly accentuated for the transfection mediated by the optimal BSA-free and BSA-coated TMZ-WRAP5/p53 complexes, and the extent of this phenomenon increases with the time of transfection. The results also demonstrated that the BSA-coated complexes were able to promote a higher decrease in U-87 cell viability when compared to the effect produced by the correspondent BSA-free complexes at 24 h (** *p* ≤ 0.001) and 48 h (* *p* ≤ 0.05). This may be in accordance with the greater p53 gene expression detected by RT-PCR for the transfection mediated by the optimal BSA-coated TMZ-WRAP5/p53 complexes (Figure 6). The difference in viability between BSA-free and BSA-coated TMZ-WRAP5/p53 complexes was, however, not statistically significant at 72 h post-transfection.

As the nanocomplexes demonstrated to be biocompatible to normal astrocyte cells (data discussed earlier in this report, Figure 3), the observed decrease in U-87 cells viability may be related to the apoptosis effect induced by p53 gene expression mediated by the nanocomplexes and by the action of TMZ. Therefore, we can correlate the obtained data on U-87 cells with efficient transfection, p53 gene expression, and apoptotic effects from the combined action of p53 and TMZ. Although a deep investigation into the synergistic effect is mandatory to unravel the cancer therapeutic effect of the optimal BSA-coated TMZ-WRAP5/p53, data from Figure 7 provide a first insight into their potential to mediate co-delivery against glioblastoma.

## 4. Conclusions

Glioblastoma therapy seeks novel and more efficacious methods to improve clinical outcomes. The combination of chemo and gene therapies could bring effective benefits. In this work, DoE was explored to rapidly and accurately reveal the most favorable BSA-coated TMZ-WRAP5/p53 gene-based plasmid DNA complexes for drug/gene co-delivery toward glioma cells. The N/P ratio and the BSA concentration were considered DoE inputs, and the size, polydispersity index, surface charge, and pDNA CC as outputs. Additionally, BSA coating time was included as a categoric input. The obtained quadratic models were statistically significant. The lack of fit was not significant; R^2^ ˃ 0.84, R^2^ adjusted ˃ 0.88, and R^2^ predicted ˃ 0.72. DoE model efficiently fitted the data, and the optimal point (experimentally validated) was revealed to be the nanocomplexes formulated at an N/P ratio of 1.03, BSA concentration of 0.08% added 25 min after formulation and exhibited a size of approximately 182 nm, a zeta potential of +9.8 mV and a pDNA CC of 96.5%. These BSA-coated TMZ-WRAP5/p53 complexes are approximately spherical in shape. Moreover, they were biocompatible with human astrocytes and stable in cell culture medium and FBS, protecting pDNA from degradation. The optimal BSA-coated TMZ-WRAP5/p53 complexes were also revealed to be hemocompatible, an important aspect of their potential in vivo applicability concerning biosafety and efficacy. Furthermore, the optimal nanocomplexes were able to gene transcription and inhibited U-87 cell viability. DoE has been demonstrated to be an extremely potent tool to unravel the most promising BSA-coated TMZ-WRAP5/p53 complexes for synergistic co-delivery in glioblastoma therapy, boosting deep in vitro research.

## Figures and Tables

**Table 1:** Composite central design and outputs for the considered inputs.

Inputs			Outputs			
Time (min)	N/P Ratio	BSA (%)	Charge (mV)	Size (nm)	PdI	pDNA CC (%)
0	0.5	0.08	−17.3	359.20	0.580	35.5
	0.5	0.18	−17.7	358.00	0.490	26.8
	0.5	0.28	−31.6	431.84	0.483	25.8
	1.0	0.08	3.8	250.82	0.378	86.4
	1.0	0.18	−1.0	307.67	0.361	80.3
	1.0	0.18	0.9	299.44	0.344	78.3
	1.0	0.18	1.2	290.97	0.381	76.7
	1.0	0.28	−3.4	308.2	0.480	86.9
	1.5	0.08	1.9	217.94	0.370	91.0
	1.5	0.18	2.6	243.96	0.310	89.0
	1.5	0.28	1.1	280.60	0.331	91.7
25	0.5	0.08	−12.4	311.25	0.391	51.2
	0.5	0.18	−18.3	324.99	0.309	39.4
	0.5	0.28	−25.7	350.13	0.276	27.0
	1.0	0.08	9.0	182.84	0.281	97.1
	1.0	0.18	5.0	211.47	0.214	91.4
	1.0	0.18	3.2	203.47	0.242	90.9
	1.0	0.18	4.5	207.74	0.234	87.2
	1.0	0.28	0.0	218.90	0.290	91.8
	1.5	0.08	11.6	134.09	0.223	95.3
	1.5	0.18	8.6	175.37	0.224	91.7
	1.5	0.28	1.3	193.59	0.228	95.8

**Table 2:** Multiple regression equations, the response surface model, and the statistical coefficients provided by the DoE analysis.

Output	Multiple Regression Equations	Model	R^2^	R^2^ Adjusted	R^2^ Predicted	Adequate Precision
Charge	+2.88 + 12.51 A − 4.58 B + 2.10 C + 2.06 AB + 0.47 AC − 0.86 BC − 9.94 A^2^ − 1.39 B^2^	Quadratic	0.9841	0.9744	0.9317	31.863
Size	+248.72 − 74.15 A + 27.26 B − 37.95 C + 1.33 AB − 6.40 AC − 4.85 BC + 33.97 A^2^ − 1.42 B^2^	Quadratic	0.9786	0.9654	0.9371	31.522
PdI	+0.30 − 0.070 A − 0.011 B − 0.073 C + 0.022 AB + 0.020 AC − 0.0056 BC + 0.019 A^2^ + 0.043 B^2^	Quadratic	0.9296	0.8863	0.7335	16.326
pDNA CC	+78.39 + 28.06 A − 3.13 B + 1.84 C + 4.39 AB − 1.53 AC − 1.71 BC − 20.55 A^2^ + 8.27 B^2^	Quadratic	0.8436	0.7474	0.7292	8.405

**Table 3:** Analysis of variance (ANOVA) results for central composite design.

Source	Charge	Size	PdI	pDNA CC
Model	<0.0001	<0.0001	<0.0001	0.0004
A	<0.0001	<0.0001	<0.0001	<0.0001
B	<0.0001	<0.0001	0.2746	0.4358
C	0.0002	<0.0001	<0.0001	0.5340
AB	0.0085	0.7898	0.0884	0.3734
AC	0.3981	0.1329	0.0626	0.7003
BC	0.1383	0.2456	0.5809	0.6682
A^2^	<0.0001	<0.0001	0.2275	0.0044
B^2^	0.1203	0.8211	0.0137	0.1903
Lack of fit	0.0961	0.0501	0.0550	0.9982

**Table 4:** Optimal point prediction and obtained mean.

Predicted Input	Output	Predicted Mean	95% CI Low for Mean	95% CI High for Mean	Obtained Mean
A- 1.03 B- 0.08% C- 25	Charge	9.7	7.2	12.2	9.8
	Size	181.5	163.0	200.0	182.3
	PdI	0.286	0.240	0.332	0.248
	pDNA CC	94.8	76.8	112.8	96.5

## Data Availability

The original contributions presented in the study are included in the article/Supplementary Material, further inquiries can be directed to the corresponding author.

## Supplementary Materials

The following supporting information can be downloaded at https://www.mdpi.com/article/10.3390/pharmaceutics16111389/s1, Figure S1: Electrophoretic analysis of BSA-coated TMZ-WRAP5/pDNA complexes at N/P ratio of 1.0 and BSA concentration ranging from 0.05% to 0.5%. Figure S2: Electrophoretic analysis of pDNA complexation capacity by the BSA-coated TMZ-WRAP5/pDNA complexes from each experiment proposed by the DoE.
