# Supplementary material for: Design of Experiments to Tailor the Potential of BSA-Coated Peptide Nanocomplexes for Temozolomide/p53 Gene Co-Delivery
Source: Pharmaceutics. 2024 Oct 29;16(11):1389. doi: 10.3390/pharmaceutics16111389 (PMC11597296; doi:10.3390/pharmaceutics16111389)
Supplement: Supplementary file 1 [file pharmaceutics-16-01389-s001.zip › pharmaceutics-3164703-supplementary.pdf]

pharmaceutics

MDPI

Supplementary Material

Design of Experiments to Tailor the Potential of BSA-Coated Peptide Nanocomplexes for Temozolomide/p53 Gene Co-Delivery

Inês Afonso, Ana R. Neves, Dalinda Eusébio, Tânia Albuquerque, Eric Vivès, Prisca Boisguérin, Adriana O. Santos, Ângela Sousa and Diana Costa

1. Results

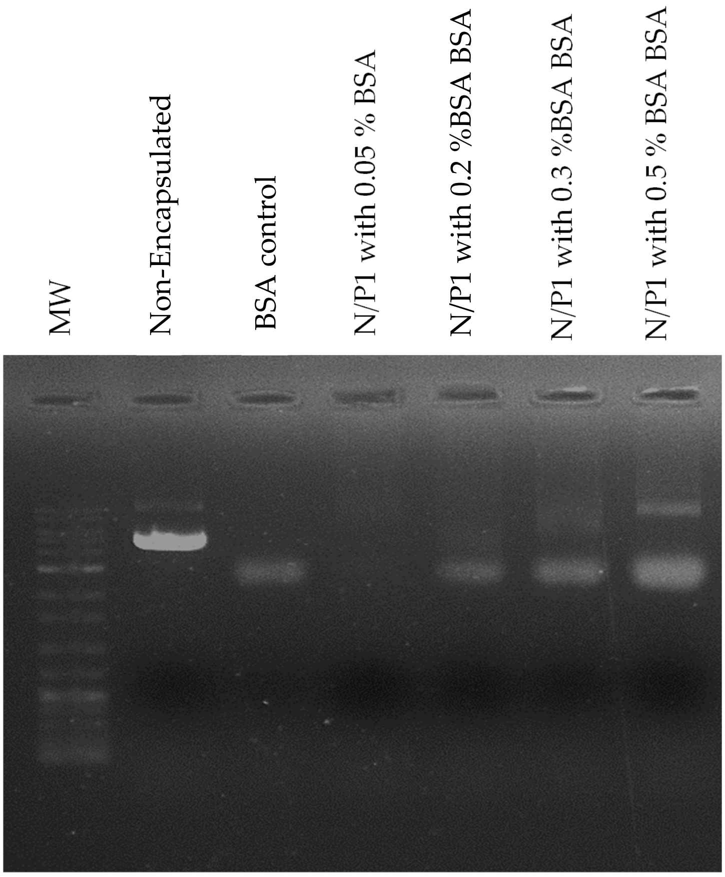

Figure S1. Electrophoretic analysis of BSA-coated TMZ-WRAP5/pDNA complexes at an N/P ratio of 1 and BSA concentration ranging from 0.05% to 0.5%. Lane 1: DNA Molecular Weight Marker; Lane 2: non-encapsulated pDNA (control); Lane 3: BSA control sample.

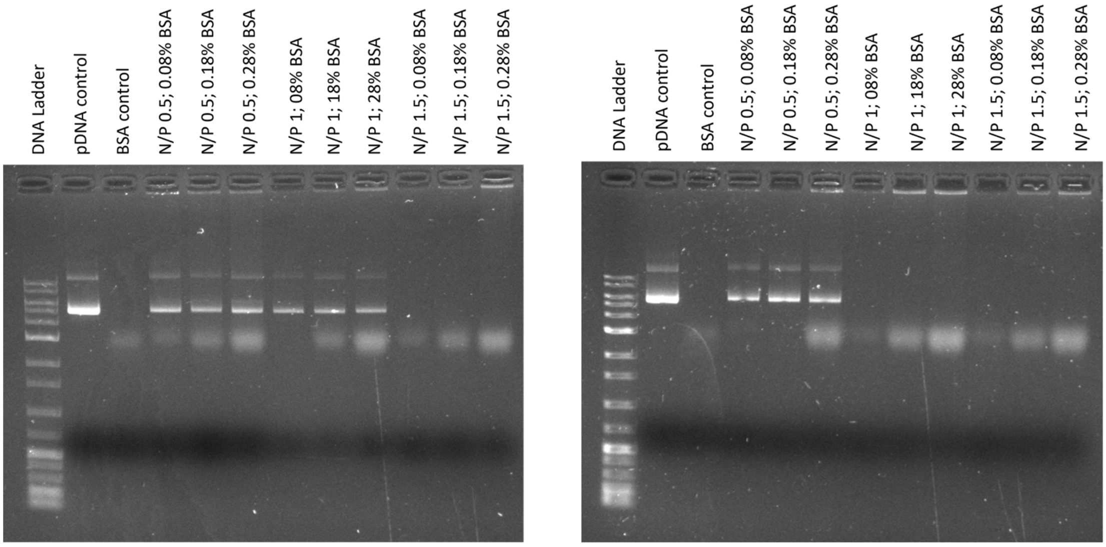

A **B**

Figure S2. Electrophoretic analysis of pDNA complexation capacity by the BSA-coated TMZ-WRAP5/pDNA complexes from each experiment proposed by DoE. A) Complexes with BSA added before formulation – 0 min; B) Complexes with BSA added 25 min after formulation. Lane 1: DNA Molecular Weight Marker; Lane 2: non-encapsulated pDNA (control); Lane 3: BSA control sample.
